# Trusted sources of information on COVID-19 vaccine in Uganda

**DOI:** 10.1186/s12911-024-02536-w

**Published:** 2024-05-23

**Authors:** Johnson Nyeko Oloya, Nelson Onira Alema, Christopher Okot, Emmanuel Olal, Eric Nzirakaindi Ikoona, Freddy Wathum Drinkwater Oyat, Baguma Steven, Denish Omoya Ochula, Patrick Odong Olwedo, Francis Pebalo Pebolo, Pamela Okot Atim, Godfrey Smart Okot, Ritah Nantale, Judith Aloyo, David Lagoro Kitara

**Affiliations:** 1 Uganda Medical Association (UMA), UMA-Acholi branch, Gulu City, Uganda; 2 Faculty of Medicine, Department of Anatomy, Gulu University, Gulu City, Uganda; 3 Gulu Regional Referral Hospital, Gulu City, Uganda; 4 Yotkom Medical Centre, Kitgum, Gulu City, Uganda; 5 ICAP at Columbia University, Freetown, Sierra Leone; 6 District Health Office, Lamwo local government, Lamwo district, Gulu City, Uganda; 7 District Health Office, Amuru local government, Amuru district, Gulu City, Uganda; 8 Moroto Regional Referral Hospital, Moroto district, Mbale City, Uganda; 9 Faculty of Medicine, Department of Reproductive Health, Gulu University, Gulu City, Uganda; 10 St. Joseph’s Hospital, Kitgum district, Gulu City, Uganda; 11 Dr. Ambrosoli Memorial HospitalKalongo, Agago district, Gulu City, Uganda; 12 Faculty of Health Sciences, Department of Public Health, Busitema University, Mbale City, Uganda; 13 Rhites-N, Acholi, Gulu City, Uganda; 14 Gulu Centre for Advanced Medical Diagnostics, Research, Trainings, and Innovations (GRUDI BIONTECH INITIATIVE), Gulu City, Uganda; 15 Faculty of Medicine, Department of Surgery, Gulu University, Gulu City, Uganda

**Keywords:** COVID-19 vaccines, Pandemic, Trusted information, Sources, Northern Uganda

## Abstract

**Background:**

The COVID-19 pandemic has dramatically impacted communities worldwide, particularly in developing countries. To successfully control the pandemic, correct information and more than 80% vaccine coverage in a population were required. However, misinformation and disinformation could impact this, thus increasing COVID-19 vaccine hesitancy in communities. Several studies observed the effect of misinformation and disinformation on COVID-19 vaccine acceptance and other responses to the pandemic in the African continent. Thus, the most trusted sources of information on COVID-19 vaccines are critical for the successful management and control of the pandemic. This study aimed to assess the most trusted sources of information on COVID-19 vaccines during the pandemic in Uganda.

**Methods:**

We conducted a cross-sectional study on 587 adult population members in northern Uganda. Single-stage stratified and systematic sampling methods were used to select participants from northern Uganda. An interviewer-administered questionnaire with an internal validity of Cronbach’s α = 0.72 was used for data collection. An Institution Review Board (IRB) approved this study and Stata version 18 was used for data analysis. A Pearson Chi-square (χ2) analysis was conducted to assess associations between trusted sources of COVID-19 vaccine information and selected independent variables. Fisher’s exact test considered associations when the cell value following cross-tabulation was < 5. A P-value < 0.05 was used as evidence for an association between trusted sources of information and independent variables. All results were presented as frequencies, proportions, Chi-square or Fisher’s exact tests, and P-values at 95% Confidence Intervals (CI).

**Results:**

In a study of 587 participants, most were males, 335(57.1%), in the age group of 25–34 years, 180(31.4%), and the most trusted source of COVID-19 vaccine information were the traditional media sources for example, Televisions, Radios, and Newspapers, 349(33.6%). There was no significant association between sex and trusted sources of COVID-19 vaccine information. However, by age-group population, COVID-19 vaccine information was significantly associated with internet use (14.7% versus 85.3%; *p* = 0.02), information from family members (9.4% versus 90.6%; *p* < 0.01), and the Government/Ministry of Health (37.9% versus 62.1%; *p* < 0.01). Between healthcare workers and non-health workers, it was significantly associated with internet use (32.2% versus 67.8%; *p* = 0.03), healthcare providers (32.5% versus 67.5%; *p* < 0.018), the Government/Ministry of Health (31.1% versus 68.9%; *p* < 0.01), and scientific articles (44.7% versus 55.3%; *p* < 0.01).

**Conclusion:**

The most trusted sources of COVID-19 vaccine information in northern Uganda were Televisions, Radios, and Newspapers. The trusted sources of COVID-19 vaccine information were not significantly different between males and females. However, there were significant differences among age groups and occupations of participants with younger age groups (≤ 44 years) and non-healthcare workers having more trust in Televisions, Radios, and Newspapers. Thus, for effective management of an epidemic, there is a need for accurate communication so that misinformation, disinformation, and malinformation in the era of “infodemic” do not disrupt the flow of correct information to communities.

**Supplementary Information:**

The online version contains supplementary material available at 10.1186/s12911-024-02536-w.

## Introduction

Globally, widespread misinformation and disinformation on the COVID-19 pandemic have been noted [1]. Misinformation is one form of information syndrome besides disinformation and malinformation [2]. Two researchers, Wardle and Derakhshan, defined *misinformation* as false information that is shared but is not necessarily intended to cause harm [2]. At the same time, *disinformation* is false information purposely created to harm a person, social group, organization, or country. In addition, *malinformation* is information based on reality and used to inflict harm on a person, organization, or country [2]. 

In many communities in the African continent, misinformation, disinformation, and malinformation have been observed from the onset of the pandemic [3]. There were some misconceptions that due to geographical conditions, for example, the warm temperatures of the African continent, the causative organism; severe-acute-respiratory-syndrome coronavirus-2(SARS-CoV-2), would not thrive [3]. Another misleading information was that Africans may have stronger immune systems to battle the virus than others [4]. Yet, a sudden rise of COVID-19 across the continent discredited all these earlier erroneous impressions [3]. 

Furthermore, COVID-19 information has been promoted extensively over mainstream and social media with limited restrictions on posts for users [5]. Moreover, many conspiracy theories have arisen globally on COVID-19 including its association with bioengineering from Wuhan, Bill Gates’s agenda on population control using vaccines, 5G technology, and many other false news such as magical cures and racist news were shared at an alarming rate, with the potential of increasing anxiety, stress, and loss of life in an era described as “infodermic” [5–7]. this misinformation related to COVID-19 in Uganda have been reported in many media outlets from the onset of the pandemic [6]. However, experts suggested that regular communications, providing updates on the status of the pandemic in-country to encourage government agencies and Task forces to use mainstream and social media to inform and provide guidance on COVID-19 situational analysis at international, national, and local levels were critical [8]. This approach was important because communities needed not to be fed on fake news and inaccurate information because these hampered progress in the control of the pandemic [9]. 

One important reference to this similar occurrence was the lessons learned from the 40-year-old experience with the successful control of HIV and AIDs in Uganda [9]. Researchers, experts, and academicians recommended that the best way of handling the HIV epidemic then, was by use of medical and non-medical interventions including regular and factual behavior change communication messages [9]. 

The success of COVID-19 pandemic control was observed in some African countries that undertook steps to control SARS-CoV-2 infection through strong leadership at the top, with proper and timely communications on simultaneous and multiple public health intervention measures [10, 11]. 

The evidence available suggests that SARS-CoV-2 transmission is most infectious in the early part, before the development of symptoms; a similar lesson was learned from the HIV epidemic in its early days when infected persons appeared normal with no symptoms and signs of AIDS as other community members [9]. 

It was suggested that biomedical and non-medical prevention strategies should be communicated to the African population as they have been proven to provide reliable protection [9]. This approach of awareness creation was recommended for adoption as transmission routes of SARS-CoV-2 were known [9]. Thus, it was proposed that with proper behavior change communications and regular updates on the virus, African Governments could adopt this approach as it has become a vital intervention for the management and control of COVID-19 [9]. 

Similarly, an online survey recently reported that 30.2% of Ugandans considered COVID-19 a disease of the white and expected mortality to be highest among white people in Europe and the USA [6]. With this information, it was evidenced that misinformation affected public perception of the risk involved and bred mistrust, which could undermine acceptance and adherence to preventive measures, including acceptance of COVID-19 vaccines. As a result, the World Health Organization (WHO), and other global and national actors responded to the threat of misinformation by running campaigns encouraging fact-checking of health information [12]. However, little was done to address COVID-19 vaccine misinformation in Uganda or to understand and address how this could influence COVID-19 vaccine acceptance in the population when vaccines became available.

Furthermore, since the COVID-19 pandemic began, many countries enacted a series of non-clinical preventive mechanisms to slow the rate of spread of SARS-CoV-2 [9–11]. However, these mechanisms could only be effective when preventive measures were correctly followed and when individuals believed the risk of COVID-19 was high enough to warrant following them [9–11]. Therefore, as the pandemic reclined and risk perceptions declined in a population, individuals were more likely to relax to follow preventive measures, and the rate of spread would increase [9–11]. 

This study aimed to assess and determine the most trusted sources of COVID-19 vaccine information in the adult population of northern Uganda during the pandemic.

## Methods

### Study design

We conducted a cross-sectional study in northern Uganda between October and November 2021. This study was part of a larger study to determine the prevalence and factors associated with compliance with the presidential directives on lockdown measures and other non-pharmacological interventions during the COVID-19 pandemic [13]. 

### Study sites

This study was conducted in the nine districts of the Acholi subregion (Gulu City, Nwoya, Omoro, Lamwo, Kitgum, Agago, Amuru, Pader and Gulu districts) [13, 14]. The Acholi subregion has just emerged from a 20-year-old civil war and it is in the post-war recovery period [13–15]. The total estimated population of the subregion is two million, three hundred thousand people in an estimated total land surface area of 28,500 km [2] [14, 16, 17]. 

### Study settings

During the study, Uganda had just eased the lockdown measures imposed as a result of the severe second wave of COVID-19 [13, 18]. At the time, the number of COVID-19 patients had significantly reduced in COVID-19 Treatment Centers (CTUs) in many health facilities in northern Uganda [13, 18]. However, health workers remained the frontline workforce (especially the nurses, midwives, cleaners, pharmacists, doctors, and laboratory staff) [13, 18, 19]. In addition, district task forces set up by the Government of Uganda along layers of administrative structures (national, districts, and communities) to support the management, prevention, and control of COVID-19 in communities met weekly to discuss new developments and plans of action [13, 18, 19]. 

Further, the President of Uganda announced new work methods in public settings, whereby only 30% of public and private institution staff were allowed physically in offices [13, 18, 19]. Thus, these COVID-19 control measures were to disrupt day-to-day contact between management, administration, and the community to interrupt the cycle of physical person-to-person contact to break the transmission cycle of COVID-19 [13, 18, 19]. 

### Study participants and sampling techniques

We interviewed five hundred and eighty-seven adult participants who were recruited by single-stage-stratified and systematic sampling techniques. Data was collected using a questionnaire that had two sections: Section A contained information on participants’ socio-demographic characteristics (age, sex, occupation, tribe, religion, district, employment status, race, highest level of education, marital status, and habits such as smoking and drinking alcohol, and comorbidities such as obesity, Asthma, heart diseases, hypertension, diabetes mellitus, and HIV and AIDs). In section B, it contained participants’ most trusted sources of information on COVID-19 vaccines. These sources of information were traditional media sources in Uganda (Televisions, Radios, and Newspapers), the Government/Ministry of Health, healthcare providers, the internet, social media (WhatsApp, Twitter, and Facebook), family members, scientific articles, pharmaceutical company reports and those who did not trust any source of information on COVID-19 vaccines (Supplementary file 1).

The participants’ selection was stratified at regional level to the nine districts of the Acholi subregion. In the districts to twenty-four health facilities where COVID-19 vaccination was administered to the general population with no pay. The twenty-four selected health facilities were (Government and non-governmental facilities) including hospitals, health centers (HC IVs), and (HC IIIs) [13, 18, 19]. 

In the selected health facilities’ outpatient departments (OPDs), we conducted a systematic sampling technique on attendants and attendees of OPDs (every third person) from the OPD registers. We defined *systematic sampling* as a probability sampling method where researchers select population members at regular intervals [20, 21]. We chose this sampling technique because it allowed us to get the desired sample size in the shortest time, thereby reducing our study team’s chances of acquiring COVID-19. Last but most importantly, a systematic sampling method helps to minimize obtaining biased samples and poor survey results in addition to eliminating clustered selection with a low probability of collecting contaminated data [20, 21]. 

### Sample size calculation

We used the Raosoft sample size calculator to determine the sample size for our study population [13, 18]. The computation was based on a 50% response distribution, 5% margin of error, and 95% Confidence Interval (CI). This online software uses a widely utilized descriptive sample size estimation formula [22, 23]. Based on the assumption of a total eligible population size of 50,000 (12.5% of the total adults above 18 years old in the Acholi subregion) in the nine districts of the Acholi subregion. The minimum sample size based on the above assumptions and factoring a 10% non-response rate is 437 participants [13, 18]. 

### Selection criteria of participants

Participants who could not speak (due to speech disability or inability to talk and not a language barrier) and were not residents in the Acholi subregion six months before the study were excluded [13, 18]. The study included consented adult outpatient attendants and attendees (≥ 18 years) in the twenty-four health facilities in the Acholi subregion during the study period.

### Data collection

Data was collected using a pre-tested questionnaire designed by the research team (supplementary file 1). The pretest was conducted in the outpatient department of Gulu Regional Referral Hospital (GRRH). The result of the pre-test was not integrated into the final data used in this analysis [13, 18]. The questions achieved an internal validity of Cronbach’s α = 0.72 [13, 18]. After obtaining informed consent from participants, an interviewer-guided questionnaire was administered to participants (in a face-to-face) in the Outpatients’ department (OPD) room, ensuring that infection, prevention, and control (IPCs) and standard operating procedures (SOPs) for COVID-19 were in place for participants and interviewers [24]. 

First, OPDs were chosen as sites for this study because they had IPCs and SOPs facilities [13, 18]. In addition, the OPD was the most convenient and preferred place to interview participants as the population had just emerged from a severe second wave of COVID-19 in Uganda [13, 18]. During the study period, the population was still in agony and apprehension due to the distress of contracting COVID-19 and were not willing to receive researchers in their offices and homes.

Second, we adopted a face-to-face questionnaire interview as the best mode of data collection despite the risks of contracting COVID-19 because we had to reach out to as many participants as possible to answer our questionnaire [13, 18]. We could have used an online approach for data collection however, previous surveys conducted in northern Uganda showed very low online and internet users (23%) [25] and mainly among persons who would not be eligible in this study because of age. Had we attempted to obtain data only from online and internet users, we would have not been able to obtain the sample size in time.

At each of the twenty-four selected health facilities, the study was conducted in the Outpatients’ Departments (OPDs), where a consented adult person (≥ 18 years) was recruited [13, 18]. The target population was attendees and attendants of the OPD services [13, 18]. A systematic sampling of every third attendant or attendee from the selected health facility’s OPD records was recruited from morning (9:00 am to 6:00 pm) every day from Monday to Saturday) until the sample size was achieved [13, 18]. 

Each interview lasted 30 to 40 min in a convenient room in the OPD. As much as the questionnaire was in English, only a few participants required translation of some questions into the local language, Acholi (5/587, 0.85%). Remarkably, only two potential participants declined to participate in the study constituting 2/589(0.34%) of the study population. Therefore, the response rate for this study was 587/589(99.7%) and most interviews went uneventfully for most participants adhering to the standard IPC and SOP guidelines [24]. 

### Ethical approval

This study was approved by the St. Mary’s Hospital, Lacor Institutional Research and Ethics Committee (LHIREC, No.0192/10/2021). In addition, it was conducted following institutional guidelines where informed consent was obtained from each participant aged (*≥* 18years) [13, 18]. Furthermore, participants’ personal information was kept confidential by excluding all personal identifiers from research documents. Also, all de-identified data were kept under lock and key throughout the study period. After the research’s completed, residual data were archived in the Faculty of Medicine of Gulu University.

### Data analysis

We analyzed this data using Stata 18 [26] and used Microsoft Excel 2019 to generate graphs. A descriptive analysis of participants’ sociodemographic and health background characteristics was conducted by presenting findings as proportions and percentages. We assessed the most trusted sources of COVID-19 vaccine information among participants and presented findings as frequencies and bar charts. The potential sources of COVID-19 vaccine information among the study population included traditional media (Televisions, Radios, and Newspapers), the Government/Ministry of Health, healthcare providers, the internet, social media, family members, scientific articles, pharmaceutical company reports, and those who did not trust any source of information on COVID-19 vaccines. From the literature on COVID-19 vaccines, we selected independent variables for example, age, sex, occupation, level of education, employment status, race, nationality, tribes, religion, districts, addresses, comorbidity, smoking and drinking status, marital status, and comorbidities for the analysis. The dependent variable was the most trusted sources of information on COVID-19 vaccines among the study population. In addition, we used Chi-square and Fisher’s exact tests to assess the factors associated with the most trusted sources of information on COVID-19 vaccines among participants. The results are reported as Chi-square tests and their respective P values and 95% Confidence Intervals (CI). We considered a p-value < 0.05 as statistically significant.

## Results

We interviewed 587 adult participants, eighteen years and above, from northern Uganda, with a questionnaire response rate of 587/589(99.7%). Only two participants, 2/589(0.34%) declined to participate, and 5/587(0.85%) required translation of the questionnaire from English to the local language, Acholi.

### The sociodemographic and health backgrounds of participants

Most participants were males,335(57.1%); in the age group of 25–34 years, 180(31.4%), and married or cohabiting, 341(58.9%). Most were Catholics, 312(53.2%); Acholi by tribe, 422(72.9%); from Gulu-Omoro districts, 220(37.5%); and had attained tertiary level of education, 261(44.5%); healthcare professionals, 136(23.2%); Ugandan by nationality, 581(99.0%); and African by race, 586(99.8%). Regarding their social behaviors, most did not use alcohol, 401(69.0%); and did not smoke cigarettes, 545(94.1%) (Table 1). On their health background information, most participants did not have comorbidities, for example, Diabetes mellitus, 16(2.7%); heart diseases, 16(2.7%); obesity, 9(1.5%); hypertension, 28(4.8%); Asthma, 15(2.6%); HIV and AIDs, 10(1.7%) and other chronic diseases, 45(7.7%). Their most trusted source of information on COVID-19 vaccines was from TV, Radio, and Newspapers, 349(33.6%)(Table 1).

### The most trusted sources of COVID-19 vaccine information among participants

**Fig. 1:**
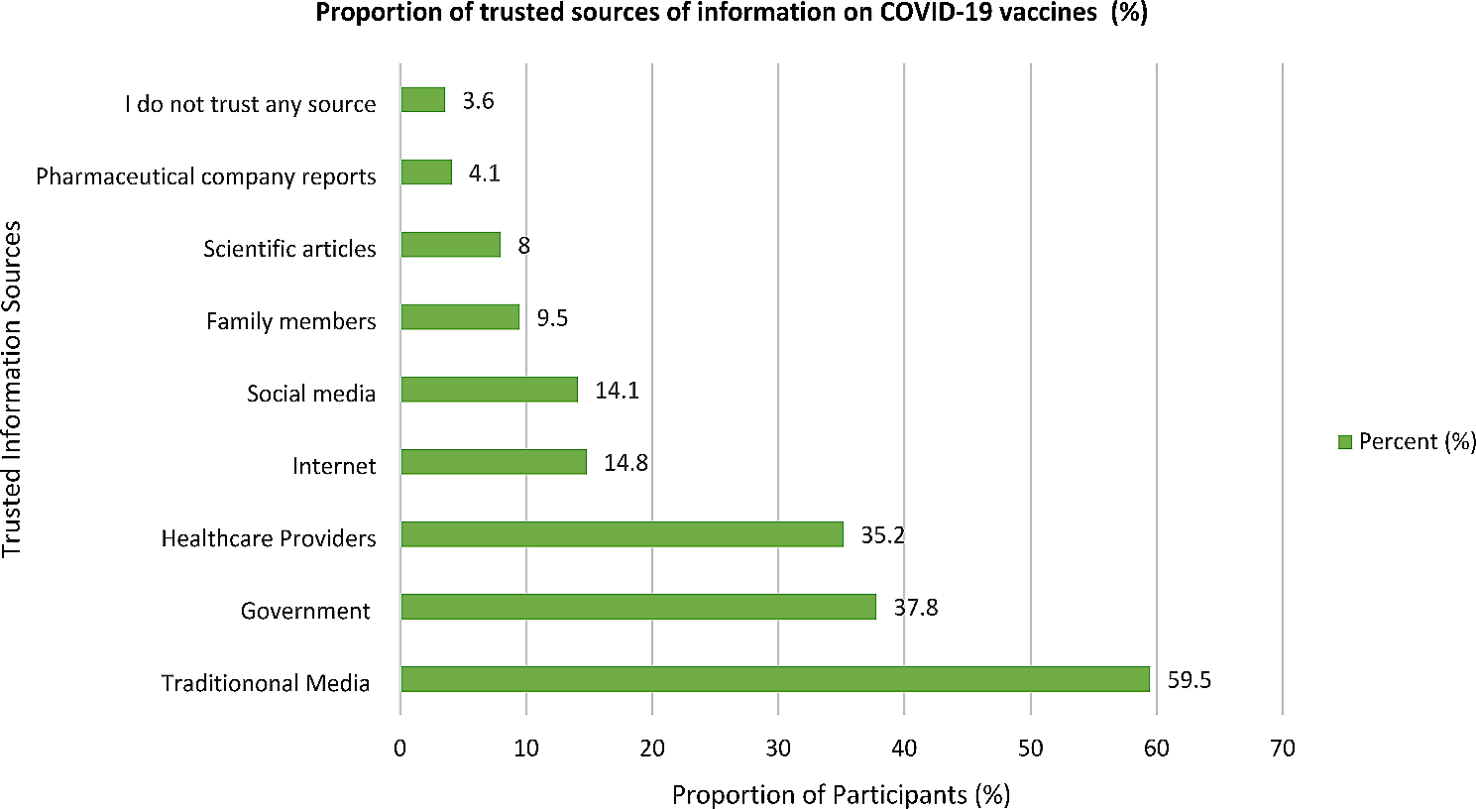
The proportion of the trusted sources of information on COVID-19 vaccines in northern Uganda

Most participants trusted traditional media sources in Uganda (Televisions, Radios, Newspapers, and websites) (59.5%) on COVID-19 vaccine information, followed by the Government/Ministry of Health (37.8%), healthcare providers (35.2%), internet (14.8%), social media (14.1%), family members (9.5%), scientific articles (8.0%), pharmaceutical company reports (4.1%), and those who did not trust any source of information on COVID-19 vaccine (3.6%) (Fig. 1).

### The preferred sources of information on COVID-19 vaccines by sex

Most y participants trusted COVID-19 vaccine information from TVs, radios, and newspapers, with females at 160(45.9%) and males, at 189(54.2%). However, most females, 219(43.8%) and males, 281(56.2%) did not trust information from internet; most females, 217(43.1%) and males, 287(56.9%) did not trust information from social media; most females, 164(43.3%) and males, 215(56.7%) did not trust information from healthcare providers; most females, 164(43.3%) and males, 201(55.1%) did not trust information from Government/MoH; most females, 232(43.7%) and males 299(56.2%) did not trust information from family members. Furthermore, most females, 242(43.0%), and males, 321(57.0%), did not trust information from pharmaceutical company reports. Also, most females, 235(43.5%), and males, 305(56.5%), did not trust information from scientific articles. In addition, most females, 243(42.9%), and males, 323(57.1%), did not trust any sources of information. Overall, there was no significant statistical difference between male and female participants in northern Uganda on the most trusted sources of information on COVID-19 vaccines (Table 2).

### Trusted sources of information on COVID-19 vaccine by age group

There were significant associations between age groups on the most trusted sources of information on COVID-19 vaccines among the study population. There was significantly less trust among the study population on COVID-19 vaccine information from the internet (14.7% versus 85.3%; *p* = 0.02), from families (9.4% versus 90.6%; *p* < 0.01), and Government/Ministry of Health (37.9% versus 62.1%; *p* < 0.01) compared to those who trusted them. Younger participants (≤ 44 years) were more pessimistic about the internet, families, and Government sources of information on COVID-19 vaccines during the pandemic (Table 3).

### Trusted sources of information on COVID-19 vaccines by occupation

The most trusted sources of information on COVID-19 vaccines among healthcare workers and non-healthcare workers were significantly different with non-healthcare workers trusting more the traditional media sources (TVs, Radios, and Newspapers) (16.3% versus 83.7%; *p* < 0.01); internet (32.2% versus 67.8%;*p* = 0.03); Healthcare providers (32.6% versus 67.5%;*p* < 0.018); Government/Ministry of Health (31.1% versus 68.9%;*p* < 0.01), and scientific articles (44.7% versus 55.3%; *p* < 0.01) compared to healthcare workers, respectively (Table 4).

**Table 1:** Sociodemographic and health background characteristics of participants from northern Uganda

Sociodemographic characteristics		*n* (%)
Sex	Female	252(42.9%)
	Male	335(57.1%)
Age in years	< 25	150(26.2%)
	25–34	180(31.4%)
	35–44	157(27.4%)
	> 44	86(15.0%)
Marital status	Married/cohabiting	341(58.9%)
	Unmarried/others	238(41.1%)
Religion	Catholic	312(53.2%)
	Protestant	245(41.7%)
	Others	30(5.1%)
Tribe	Acholi	425(72.9%)
	Lango	41(7.0%)
	Others	117(20.1%)
Districts	Gulu-Omoro	220(37.5%)
	Kitgum-Lamwo	133(22.7%)
	Amuru-Nwoya	92(15.7%)
	Agago-Pader	86(14.7%)
	Others	56(9.5%)
Levels of formal education	Tertiary	261(44.5%)
	Secondary	225(38.3%)
	Primary	64(10.9%)
	None	37(6.3%)
Occupations	Health professional	136(23.2%)
	Agriculture/self-employed	115(19.6%)
	Employed/retired	82(14.0%)
	Student/unemployed	105(17.9%)
	Others	149(25.4%)
Nationality	Ugandan	581(99.0%)
	non-Ugandan	6(1.0%)
Race	African	586(99.8%)
	Caucasian	1(0.2%)
**Health background information**		
Alcohol use	No	401(69.0%)
	Yes	180(31.0%)
Smoking status	No	545(94.1%)
	Yes	34(5.9%)
Diabetes Mellitus	No	571(97.3%)
	Yes	16(2.7%)
Heart diseases	No	571(97.3%)
	Yes	16(2.7%)
Obesity	No	578(98.5%)
	Yes	9(1.5%)
Hypertension	No	559(95.2%)
	Yes	28(4.8%)
Asthma	No	572(97.4%)
	Yes	15(2.6%)
HIV	No	577(98.3%)
	Yes	10(1.7%)
Other chronic diseases	No	542(92.3%)
	Yes	45(7.7%)
What are your most trusted sources of information on COVID-19 vaccines?	TVs, Radios, and Newspapers	349(33.6%)
	Internet	87(8.4%)
	Social Media	83(8.0%)
	Healthcare practitioners	206(19.8%)
	The Government/MoH	222(21.4%)
	Family members	56(19.8%)
	Pharmaceutical company reports	24(2.4%)
	Scientific articles	47(4.5%)
	No trusted source of information	21(2.0%)

MoH = Ministry of Health

**Table 2:** The preferred sources of information on COVID-19 vaccines by sex of participants

Dependent variables		Sex (*n* = 587)		Χ² P-value
		Female (%)	Male (%)	
TV, Radios, and Newspapers	No	92(38.7%)	146(61.3%)	0.08
	Yes	160(45.9%)	189(54.2%)	
Internet	No	219(43.8%)	281(56.2%)	0.31
	Yes	33(37.9%)	54(62.1%)	
Social Media	No	217(43.1%)	287(56.9%)	0.88
	Yes	35(42.2%)	48(57.8%)	
Health care workers	No	164(43.3%)	215(56.7%)	0.90
	Yes	88(42.7%)	118(57.3%)	
Family members	No	232(43.7%)	299(56.3%)	0.25
	Yes	20(35.7%)	36(64.3%)	
Government/Ministry of Health	No	164(44.9%)	201(55.1%)	0.21
	Yes	88(39.6%)	134(60.4%)	
Pharmaceutical company reports	No	242(43.0%)	321(57.0%)	0.90
	Yes	10(41.7%)	14(58.3%)	
Scientific articles	No	235(43.5%)	305(56.5%)	0.33
	Yes	17(36.2%)	30(63.8%)	
No trusted source	No	243(42.9%)	323(57.1%)	0.99
	Yes	9(42.9%)	12(57.1%)	

**Table 3:** Trusted sources of information on COVID-19 vaccines by age-group of participants

Dependent variables		Ages (years) n, (%)				Χ² P-value
		< 25	25–34	35–44	> 44	
Traditional Media (TVs, Radios, and Newspapers)	No	58(24.9%)	77(33.1%)	64(27.5%)	34(14.6%)	0.89
	Yes	92(27.1%)	103(30.3%)	93(27.4%)	52(15.3%)	
Internet	No	135(27.6%)	142(29.0%)	139(28.4%)	73(14.9%)	0.02*
	Yes	15(17.9%)	38(45.2%)	18(21.4%)	13(15.5%)	
Social Media	No	131(26.6%)	146(29.7%)	135(27.4%)	80(16.3%)	0.06
	Yes	19(23.5%)	34(41.9%)	22(22.2%)	6(7.4%)	
Health care workers	No	100(27.0%)	116(31.4%)	105(28.4%)	49(13.2%)	0.37
	Yes	50(24.9%)	64(31.8%)	50(24.9%)	37(18.4%)	
Family members	No	141(27.2%)	173(33.3%)	133(25.6%)	72(13.9%)	< 0.01*
	Yes	9(16.7%)	7(12.9%)	24(44.4%)	14(25.9%)	
Government / Ministry of Health	No	109(30.6%)	119(33.4%)	81(22.8%)	47(13.2%)	< 0.01*
	Yes	41(18.9%)	61(28.1%)	76(35.0%)	39(17.9%)	
Pharmaceutical company reports	No	145(26.4%)	174(31.7%)	150(27.3%)	80(14.6%)	0.51
	Yes	5(20.8%)	6(25.0%)	7(29.2%)	6(25.0%)	
Scientific articles	No	143(27.1%)	167(31.7%)	138(26.2%)	79(14.9%)	0.11
	Yes	7(15.2%)	13(28.3%)	19(41.3%)	7(15.2%)	
I do not trust any source.	No	143(25.9%)	173(31.3%)	152(27.5%)	84(15.2%)	0.80
	Yes	7(33.3%)	7(33.3%)	5(23.8%)	2(9.5%)	

*significant P value

**Table 4:** Trusted sources of information on COVID-19 vaccines by occupation of participants

Dependent Variables		Occupation n (%)		Χ² P-value
		Health workers	Non-health workers	
Traditional Media (TV, radios, Newspaper)	No	79(33.2%)	159(66.8%)	< 0.01*
	Yes	57(16.3%)	292(83.7%)	
Internet	No	108(21.6%)	392(78.4%)	0.03*
	Yes	28(32.2%)	59(67.8%)	
Social Media	No	110(21.8%)	394(78.2%)	0.06
	Yes	26(31.3%)	57(68.7%)	
Healthcare providers	No	69(18.2%)	310(81.8%)	< 0.018*
	Yes	67(32.5%)	139(67.5%)	
Family members	No	123(23.2%)	408(76.8%)	0.99
	Yes	13(23.2%)	43(76.8%)	
Government / Ministry of Health	No	67(18.4%)	298(81.6%)	< 0.01*
	Yes	69(31.1%)	153(68.9%)	
Pharmaceutical company reports	No	130(23.1%)	433(76.9%)	< 0.83
	Yes	6(25.0%)	18(75.0%)	
Scientific articles	No	115(21.3%)	425(78.7%)	< 0.01*
	Yes	21(44.7%)	26(55.3%)	
I do not trust any source	No	133(23.5%)	433(76.5%)	0.44
	Yes	3(14.3%)	18(85.7%)	

*significant P value

## Discussion

The most substantial finding from this study was that most participants from northern Uganda considered traditional media sources (Televisions, Radios, and Newspapers) as the most trusted sources of information on COVID-19 vaccines (Table 1; Fig. 1). As shown in many publications, there have been uncertainties about the COVID-19 vaccine rollout in many communities in Uganda, mainly because of misinformation, disinformation, and malinformation circulating in many media sources that are not properly regulated [27–31]. Fortunately, this study found that most participants trusted the traditional media sources on COVID-19 vaccines such as Televisions, Radios, and Newspapers that are regulated by the Government of Uganda [27–31]. This may explain why COVID-19 vaccine acceptance among the population of northern Uganda has been high compared to other regions of Uganda [31]. Experts argue that the flow of factual and correct information on COVID-19 vaccines is critical for the mobilization and engagement of the population on COVID-19 vaccine acceptance, which is crucial for the management and control of the pandemic [29, 30]. In addition, misinformation, disinformation, and malinformation are significant hindrances to successfully managing any epidemic.27–31 Thus, the need to consistently update the population on correct and factual information remains critical for successfully controlling and managing any epidemic [27–31]. 

### Trusted sources of information on COVID-19 vaccines by participants

Our study found that, in general, the most trusted sources of COVID-19 vaccine information among participants were the traditional media sources (Television, Radios, and Newspapers) (59.5%), followed by the Government/Ministry of Health (37.8%), healthcare providers (35.2%), internet (14.8%), social media (14.1%), family members (9.5%), scientific articles (8.0%), pharmaceutical company reports (4.1%), and least among those who did not trust any sources of information on COVID-19 vaccines (3.6%) (Fig. 1). This finding is significant as health planners and managers in Uganda could use this information to plan and execute future interventions to control and manage any emerging epidemic of this magnitude. This finding was consistent with other studies conducted in the African continent [27, 28, 32, 33] and others worldwide [34–37]. 

### Trusted sources of information on COVID-19 vaccines by age groups

Our analysis revealed that there were varying differences across age groups on the most trusted sources of COVID-19 vaccine information except for sources such as; the internet, family, and government/Ministry of Health where most participants did not trust their information. Younger participants (< 45 years) were less likely to report the internet, family, and Government/MOH as the most trusted sources of COVID-19 vaccine information compared to older adults (> 45 years). Furthermore, there have been significant associations between younger age groups (≤ 44 years) and the most trusted sources of information on COVID-19 vaccines, with most younger age groups having less trust in information sources from the internet, family, and Government/Ministry of Health. In this, there was significantly less trust in COVID-19 vaccine information from the internet (14.7% versus 85.3%;*p* = 0.02), from families (9.4% versus 90.6%; *p* < 0.01), and Government/Ministry of Health (37.9% versus 62.1%; *p* < 0.01) compared to those who trusted them (Table 3). We, the authors argue that these findings could be used positively by strategists and planners of healthcare systems in Uganda to support a successful rollout of COVID-19 vaccines in Uganda. Social media and the internet were sources of COVID-19 vaccine information to the general population but relatively more expensive and difficult to access and more prone to misinformation, disinformation, and malinformation [31, 38]. 

### Trusted sources of information on COVID-19 vaccines by occupation

Our study found that the most trusted sources of information on COVID-19 vaccines between healthcare workers and non-health workers differed significantly. Non-health workers trusted traditional media sources (TVs, Radios, Newspapers) more than health workers. For example, the most trusted sources of COVID-19 vaccine information among non-healthcare workers were traditional media sources (83.7% versus 16.3%; *p* < 0.01); internet (67.8% versus 32.2%; *p* = 0.03); healthcare providers (67.5% versus 32.6%; *p* < 0.018); Government/Ministry of Health (68.9% versus 31.1%; *p* < 0.01), and scientific articles (55.3% versus 44.7%; *p* < 0.01) than healthcare workers, respectively (Table 4). This finding is interesting as the general population would expect healthcare workers to be more receptive to information on COVID-19 vaccines, as this would help them to sensitize and mobilize the population on the rollout of COVID-19 vaccines for its management and control in the population. This finding contrasts many studies in Uganda and elsewhere [30–32]. 

With regards to healthcare workers, the most frequently selected sources of information on COVID-19 vaccines were the Government/Ministry of Health, followed by healthcare providers, traditional media (TVs, Radios, and Newspapers), internet, social media, family members, and pharmaceutical reports in the descending order, respectively (Table 3). However, the most trusted sources of information on COVID-19 vaccines among non-health workers were traditional mass media/news media websites, followed by messages from the Ugandan Ministry of Health and the healthcare providers (Table 3). Health workers also consulted WHO information for guidance on COVID-19 vaccines and scientific articles, but the numbers were less compared to non-health workers (Table 3). This flow of COVID-19 vaccine information must be improved in the following ways: improvements in the content and format of information, increased training and learning opportunities, improvements in dissemination strategies, and empowerment of health workers [39]. 

Remarkably, this study found that most participants in northern Uganda had confidence in traditional media sources as the most trusted sources of information on COVID-19 vaccines, and this could be used by Ugandan Ministry of Health planners to strategize on how to reach out to the population for the control of any emerging epidemic in Uganda. These results suggest that plans to promote factual and accurate information flow on COVID-19 vaccines must take a dual focus: working with communities and influential leaders in the said communities and analyzing patterns of use and access to the different media sources. Further, qualitative research should be conducted to identify how the most trusted sources of information on COVID-19 were interpreted and spread through community networks.

### Strengths and limitations of this study

This study had many strengths: First, it was conducted on a determined sample size with a high power (> 80%) among community members in northern Uganda, so most information obtained could be generalizable. Second, we used a systematic sampling method, which is a probability sampling method, and thus, the information obtained can represent findings in a specific location of similar settings. Third, the information obtained helps inform policy on the dissemination of health-related matters to the population.

However, this study had some limitations: First, the nature of the study design is cross-sectional with inherent limitations of not measuring variables over time, thus risking the ability to capture the dynamism of changing times and perceptions of participants. Second, we captured the views and opinions of adult participants ≥ 18 years old, yet most of the population in northern Uganda was below 18 years old [11]. This presents a challenge of representation bias among younger age groups and may become problematic when designing strategies for preventing and controlling such diseases in future outbreaks. Third, our findings that most participants in our study had attained tertiary level of education pose a challenge of representation as information obtained from studies in northern Uganda show that most of the population do not have tertiary education [11]. This finding may present a selection bias of our study population.

### Generalizability of results

These findings may be generalized among rural communities in sub-Saharan African countries with similar contexts.

## Conclusion

The most trusted sources of COVID-19 vaccine information in northern Uganda were Televisions, Radios, and Newspapers. The trusted sources of COVID-19 vaccine information were not significantly different between males and females. However, there were significant differences among age groups and occupations of participants with younger age groups and non-healthcare workers having more trust in Televisions, Radios, and Newspapers. Thus, for effective management of an epidemic, there is a need for accurate communication so that misinformation, disinformation, and malinformation in the era of “infodemic” do not disrupt the flow of correct information to communities.

### Electronic supplementary material

Below is the link to the electronic supplementary material.

Supplementary Material 1

## Data Availability

All datasets supporting this article’s conclusion are within this paper and are accessible by a reasonable request to the corresponding author.

## Declarations

### Authors’ information

Dr. Johnson Nyeko Oloya (JNO) is a medical officer at Moroto Regional Referral Hospital, Moroto district, Uganda; Dr. Nelson Onira Alema (NOA) is a lecturer at Gulu University, Faculty of Medicine, Department of Anatomy, Gulu City, Uganda; Dr. Christopher Okot (CO) is a medical officer Special Grade at Gulu Regional Referral Hospital, Gulu City, Uganda; Dr. Emmanuel Olal (EO) is a Public Health Specialist and a Physician at Yotkom Medical Centre in Kitgum district, Kitgum, Uganda; Dr. Eric Nzirakaindi Ikoona (ENI) is a Technical Director at ICAP at the University of Columbia, Sierra Leone, Liberia; Dr. Freddy Wathum Drinkwater Oyat (FWDO) is a senior physician, a public health specialist, and a member of Uganda Medical Association, UMA-Acholi branch, Gulu City, Uganda; Dr. Steven Baguma (SB) is a medical officer at Gulu Regional Referral Hospital, Gulu City, Uganda; Dr. Denish Omoya Ochula (DOO) is a District Health officer at Lamwo district local government, Lamwo, Uganda; Dr. Patrick Olwedo Odong (POO) is a District Health Officer at Amuru local Government, Amuru district, Uganda; Dr. Francis Pebalo Pebolo (FPP) is a lecturer at Gulu University, Faculty of Medicine, Department of Reproductive Health, Gulu City, Uganda; Dr. Pamela Okot Atim (POA) is a senior Obstetrician and Gynecologist and a Medical Superintendent at St. Joseph’s Hospital, Kitgum, Uganda; Dr. Geoffrey Smart Okot (GSO) is a senior Surgeon and a Medical Superintendent at Dr. Ambrosoli Memorial Hospital, Kalongo, Agago district, Uganda; Dr. Ritah Nantale (RN) is a Research Manager for the Centre of Excellence for Maternal, Reproductive and Child Health at Busitema University, Mbale City, Uganda; Dr. Judith Aloyo (JA) is a Deputy Chief of Party at Rhites-N, Acholi, Gulu City, Uganda; Prof. David Lagoro Kitara (DLK) is a Takemi fellow of Harvard University and a Professor at Gulu University, Faculty of Medicine, Department of Surgery, Gulu City, Uganda.

## Abbreviations

COVID-19: Coronavirus disease-19
CTU: Coronavirus Treatment Unit
HC: Health Centre
IRB: Institutional Review Board
OPD: Outpatient Department
SARS-CoV-2: Severe Acute Respiratory Syndrome coronavirus-2
WHO: World Health Organization
