# Supplementary material for: Trusted sources of information on COVID-19 vaccine in Uganda
Source: BMC Med Inform Decis Mak. 2024 May 23;24:132. doi: 10.1186/s12911-024-02536-w (PMC11112838; doi:10.1186/s12911-024-02536-w)
Supplement: Supplementary file 1 — Supplementary Material 1 [file 12911_2024_2536_MOESM1_ESM.docx]

**Supplementary file I: Questionnaire**

| **Socio-demographics characteristics of participants** | |
| --- | --- |
| Sex | 🞎 Male 🞎 Female |
| Age (years) | **…………………………………………..** |
| Marital status | 🞎 Single 🞎 Married 🞎 Divorced 🞎 Others |
| Religion | 🞎 Catholic 🞎 Protestant 🞎 Muslim 🞎 Born Again  🞎 Others, Specify………………………………………………………………. |
| Tribe | 🞎 Acholi 🞎 Lango 🞎 Muganda 🞎 Itesot 🞎 Madi  🞎 Others, Specify………………………………………………………………. |
| District | 🞎 Gulu 🞎 Kitgum 🞎 Pader 🞎 Lamwo 🞎 Amuru  🞎Others, Specify………….……………………………………………………. |
| Highest level of education attained | 🞎 No education 🞎 Primary education  🞎Secondary education 🞎 Graduate 🞎 Postgraduate |
| Employment status | 🞎 Employed 🞎 Unemployed 🞎 Retired.  🞎Others, Specify ……………………………………………………………… |
| Current occupation | 🞎 Specify………………………………………………………………………….. |
| Nationality | 🞎 Ugandan  🞎 non-Ugandan; Specify…………………………………………………… |
| Race | 🞎 African 🞎 Asian  🞎 Caucasian 🞎 Others, Specify……………………………………… |
| Where do you live? | 🞎 District…………………….🞎 Sub County…….…………………………  🞎 Constituency……………🞎 Village, Specify………………………… |
| Do you drink alcohol? | 🞎 Yes 🞎 No 🞎 Never drunk alcohol in my lifetime |
| Do you smoke cigarettes? | 🞎 Yes 🞎 No 🞎 Never smoked in my lifetime |
| **Medical History** | |
| What medical conditions do you currently have? | 🞎 Diabetes 🞎 Hypertension  🞎 Heart Disease 🞎 Asthma  🞎 Obesity 🞎 HIV  🞎 Others Specify…………………………………………………………….. |
| **Perceptions towards lockdown policy and wearing of facemasks** | |
| In general, did you agree with the Presidential directive on lockdown of the country because of COVID-19 in June 2021? | 🞎 Yes  🞎 No  If no, why not?...................................................................... |
| **Questions on COVID-19 pandemic in Uganda** | |
| What are you most worried about during this COVID-19 pandemic? ***(Tick not more than 5)*** | 🞎 Fear of becoming infected myself  🞎 Fear of a family member becoming infected  🞎 Death  🞎 Financial related worries  🞎 Job-related worries  🞎 Food insecurity related worries  🞎 Unavailability of vaccines  🞎 Being a plot or conspiracy theory  🞎 Being forced to take a medication  🞎 Being forced to take a vaccine  🞎 I am not worried about any issues  🞎 Others, specify………………………………………………………………. |
| What do you understand by social/physical distancing in COVID-19? | 🞎 Keeping 2 meters away from any person  🞎 Keeping your phone away from people  🞎 Locking yourself in your own room  🞎 Keeping visitors away from your home  🞎 Keeping in isolation even in your home |
| Is social/physical distancing important in curbing the spread of COVID-19? | 🞎 Yes 🞎 No 🞎 I do not know |
| Did you obey the Presidential orders on COVID-19 lockdown of Uganda in June 2021? | 🞎 Yes 🞎 No |
| **Perceptions to COVID-19 Lockdown and use of facemasks** | |
| What factors helped you to go through the lockdown period in the year 2021? | 🞎 Availability of the major needs  🞎 Availability of food and money  🞎 My desire to help prevent the transmission of the virus  🞎 My resolve to avoid contact with an infected person  🞎 My fear of contracting the virus  🞎Others, specify………………………………………………………………. |
| What are your major fears about the COVID-19 pandemic? | 🞎 Contracting the virus  🞎 Death  🞎 infectious nature of the virus  🞎 Non-compliance of the Public  🞎 Asymptomatic carriers  🞎 Others, specify………………………………………………………………. |
| Which four items do you recommend as the most useful methods for prevention and control of COVID-19 pandemic in your community? | 🞎 Vaccination against COVID-19  🞎 Use of facemasks and sanitizers  🞎 Enforce-stay-at home orders  🞎 Discover the cure for the disease  🞎 Compulsory testing of all Ugandans for COVID-19  🞎 Sensitization and mobilization of the population |
| What is wearing of facemask for COVID-19? | 🞎 Covering the mouth, nose, and eyes  🞎 Covering the mouth, nose, and ears  🞎 Covering the mouth and nose  🞎 Covering the mouth, nose, ears, and eyes  🞎 Covering the mouth only |
| In what three circumstances are you most required to use your facemasks during this COVID-19 pandemic? | 🞎 When in public  🞎 When in the Supermarket  🞎 When in my office  🞎 While jogging in the outdoor circumstance  🞎 When at my home  🞎 I do not know |
| Have you been wearing facemasks while in public during this COVID-19 pandemic? | 🞎 Yes 🞎 No 🞎 I do not remember |
| What challenges did you face while wearing your facemask? | 🞎 I feel like suffocating.  🞎 It is very inconveniencing.  🞎 Masks take away my beauty.  🞎 It does not help with the COVID-19 disease.  🞎 Others, specify………………………………………………………………. |
| Did you follow the Presidential directive on wearing facemasks during this pandemic? | 🞎 Yes 🞎 NO  🞎 If No, why?......................................................................... |
| Is there any importance in wearing facemasks in curbing the spread of COVID-19 pandemic? | 🞎 Yes 🞎 No 🞎 I do not know |
| What is your most trusted source of information on social distancing during the COVID-19 pandemic? | 🞎 Traditional Media (TVs, Radios, Newspapers)  🞎 Internet  🞎 Social media (Facebook, Twitter, WhatsApp etc.)  🞎 Healthcare providers: Physicians, pharmacists, etc.  🞎 Family-members  🞎 Government (MoH)  🞎 The pharmaceutical company reports.  🞎 Scientific articles  🞎 I do not trust any source |
